# The Survivor: A Clinical Case of Tetanus in a Non-Immunized, Parenteral Drug User, Former Female Convict with HIV and HCV

**DOI:** 10.3390/vaccines8020308

**Published:** 2020-06-17

**Authors:** Nancy Vicente-Alcalde, Tamara Martín-Casquero, Esther Ruescas-Escolano, José Tuells

**Affiliations:** 1 Penitentiary Center Alicante II, Villena, Alicante, Carretera N-330, Km. 66, 03400 Villena, Spain; nanvical@gmail.com; 2 Servicio de Urgencias, Hospital Universitario del Vinalopó-Elche, Calle Tonico Sansano Mora, 03293 Elche, Spain; tmartincasquero@gmail.com (T.M.-C.); esther.ruescas@gmail.com (E.R.-E.); 3 Department of Community Nursing, Preventive Medicine and Public Health and History of Science, University of Alicante, San Vicente del Raspeig, 03690 Alicante, Spain

**Keywords:** tetanus, vaccine, injecting drug user (IDU), Human Immunodeficiency Virus (HIV), Hepatitis C Virus (HCV), prisoner, Spain

## Abstract

Tetanus is a serious and potentially fatal systemic disease, caused by the bacterium *Clostridium tetani*. It is estimated that 1 million cases occur worldwide annually. Immunization programs have drastically decreased tetanus in developed countries, and the sporadic cases have been linked to injecting drug users (IDUs), immunosuppressed individuals, or those older than 65 without complete vaccination. Regrettably, it is still endemic in under-developed countries. In Spain, an average of 10 cases are notified each year, especially affecting those older than 65. The present article describes a case of a 48-year-old Spanish woman, an ex-convict and IDU with infection antecedents of Human Immunodeficiency Virus (HIV) and Hepatitis C (HCV), who was admitted to the Emergency Department of a University Hospital with cervical rigidity and trismus. In a few hours, a neurological and respiratory deterioration was observed, resulting in admittance to the intensive care unit under the suspicion of a generalized tetanus infection. The treatment included hemodynamic support and respiratory therapy, antibiotics, muscle relaxants, sedatives, and tetanus immunoglobulin. Her stay in the ICU lasted 47 days. The clinical suspicion, the antecedents of risk, and the verification of the vaccination records should provide early guidance for diagnostics and the establishment of a treatment in these cases.

## 1. Introduction

Tetanus is an infectious disease caused by the bacteria *Clostridium tetani*, and is preventable through active immunization. These bacteria are specifically found in soil, ashes, animal excrements, and rusty tools, and are highly resistant to heat and most antiseptic products [1,2]. The spores can remain inactive in the soil and can continue to be infectious for more than 40 years. The contamination from spores is very frequent, but the production of the toxin only occurs in wounds with greater tissue necrosis (lacerations, puncture wounds, burns, diabetic ulcers, etc.) [3]. The disease started to be fought after the isolation of the toxin by Arthur Nicolaier in 1884 and the posterior contributions of two researchers, Emil von Behring and Kitasato Shibasaburo from the Department of Infectious Diseases at the Robert Koch Institute in Berlin, who discovered the tetanus toxoid [4]. 

In the adult population, the risk of suffering from the disease is closely linked to injecting drug users (IDUs), and the elderly who are unvaccinated or partially vaccinated. The infections related to the IDUs continue to be a substantial burden on healthcare systems around the world [5,6,7]. The symptomology from tetanus is well documented, and in it observed a state of permanent motor activation that promotes the appearance of involuntary muscle spasms. It has an elevated mortality (from 20–45% of the infected) and a low morbidity. The illness can be developed in any geographical area or age group. The protection against tetanus is achieved through immunization, either active (tetanus vaccine), available through worldwide programs of infant vaccination, or passive (specific tetanus immunoglobulin). Despite the existence of the vaccine with the tetanus toxoid, tetanus is still an endemic disease in under-developed countries, and its incidence tends to increase after natural disasters. In these countries, it is a problem for public health due to the mortality associated with neonatal tetanus [8,9].

Here, we describe the case of a patient who arrived at our hospital with a clinical profile whose progression confirmed the suspicion of a tetanus infection. The comorbidities and risk factors, as well as the changes and treatment, are analyzed below.

## 2. Case 

The case involves a 48-year-old woman who visited the Emergency Department of a University Hospital from the Community of Valencia, Spain, due to cervical rigidity and difficulty in opening her mouth. The patient recounted suffering a fall 48 h before, with a strong contusion on her back that resulted in a cervical cramp, initially treated with an anti-inflammatory cream. After 24 h, the generalized rigidity started in her upper limbs and trunk, with increasing difficulties in opening the mouth, the latter being the reason for the hospital visit. She had a normal level of awareness and an acceptable ability to communicate.

Initially, a craniocervical CT scan was performed without finding anything important. She was later evaluated by the maxillofacial surgery staff, who diagnosed a post-traumatic arthritis of the temporomandibular joint. The patient was admitted to the Area of Observation.

### 2.1. Personal Antecedents

Among her antecedents, the following were highlighted: being a parenteral drug user (heroin and others) and different admissions (between 2009 and 2018) in penitentiary institutions due to minor offences. She had been diagnosed with HIV and HCV with irregular monitoring and had been treated with Kivexa^®^ (Abacavir/Lamivudina), Reyataz^®^ (Atozanavir), Norvir^®^ (Ritonavir), Prisdal^®^ (Citalopram), Lexatin^®^ (Bromazepan), and Loramet^®^ (Lormetazepam). Likewise, she had been previously admitted to the Internal Medicine Unit due to abscesses and cellulitis of the soft tissue in the venipuncture areas (in 2012). The only surgery antecedent was a tubal ligation after having seven children.

### 2.2. Physical Check-Up and Clinical Evolution

At the time of admission into the emergency’s unit, she was conscious and could focus, although she had a bad general state and was malnourished, but well-hydrated. She had a fever of 38 °C, adequate peripheral perfusion, a blood pressure of 106/79 mmHg, and regular tachycardia of 130 beats per minute (bpm) without murmurs. She was eupneic with an abundantly dispersed bilateral rhonchus. The abdomen was normal and did not have edemas or signs of deep vein thrombosis in her lower extremities. Numerous lesions due to venipunctures were evident in her four limbs, with cutaneous erosions from the different puncture events. The cervical rigidity was evident, without the possibility of full extension, with a limited buccal opening and facial spasms with exposure of teeth compatible with risus sardonicus.

In the following hours, there was rapid respiratory deterioration, with a high increase of secretions in the airway and bronchoaspiration, which was confirmed by chest radiography, showing bibasal infiltrates. The progression resulted in a situation of acute respiratory insufficiency. The secretions were aspirated through the nasal cavity due to the difficulty in opening the mouth. A non-invasive mechanical ventilation (NIMV) was performed in BiPAP mode with a pressure of 20/8 and 100% oxygen supply. The condition of the patient worsened in three aspects: deterioration of consciousness to Glasgow 3; painful muscle spasms interpreted as tonic–clonic movements together with opisthotonos; and desaturation despite NIMV (arterial blood gas at pH 6.9 and pCO_2_ 97). The persistence of fever, sinus tachycardia, hypotension, profuse sweating, neck rigidness, and trismus was also observed.

The patient was admitted to the Intensive Care Unit (ICU), where an endotracheal intubation was performed, as well as a spinal tap, which ruled out meningoencephalitis. The National Vaccine Registry was checked, and it was verified that the patient had not been immunized against tetanus. With these data, and faced with the possibility of generalized severe tetanus, a dose of anti-tetanus gammaglobulin was administered immediately, the tetanus vaccine was started, and metronidazole was prescribed for 10 days. She was admitted to an ICU room with acoustic isolation, and tactile stimuli were avoided.

On the 6th day at the ICU, an early percutaneous tracheostomy was performed in anticipation of prolonged mechanical ventilation. During the first three weeks, the patient was in a severe comatose state with persistent muscle spasms and autonomic instability, needing deep sedation and hemodynamic and respiratory support, which were adjusted according to the intensity of the symptoms. After 14 days in the ICU, a second dose of the anti-tetanus gammaglobulin was administered.

During her stay at the ICU, she was cared for by an infectious diseases specialist, who provided advice on the use of antiretroviral drugs due to her chronic pathology (HIV), as well as on antibiotics according to the results of the cultures (see Table 1). She was also evaluated by the ophthalmology unit due to the appearance of corneal ulcers due to environmental exposure. She was treated by the rehabilitation unit early on, with the use of passive kinesiotherapy as a treatment against spasticity. Lastly, she also received care from a psychiatrist, who started an anti-depression treatment after assessing the emotional instability that appeared after the start of recovery.

Starting on the third week, the spasms decreased, and sedation and relaxation were gradually reduced. Once respiratory and hemodynamic stability was reached, the support treatments were withdrawn with a good response. After 47 days in the ICU, she was moved to the internal medicine unit, where she completed her recovery. The hospital discharge was given with motor recovery ad integrum.

### 2.3. Complementary Tests and Monitoring

**Figure 1:**
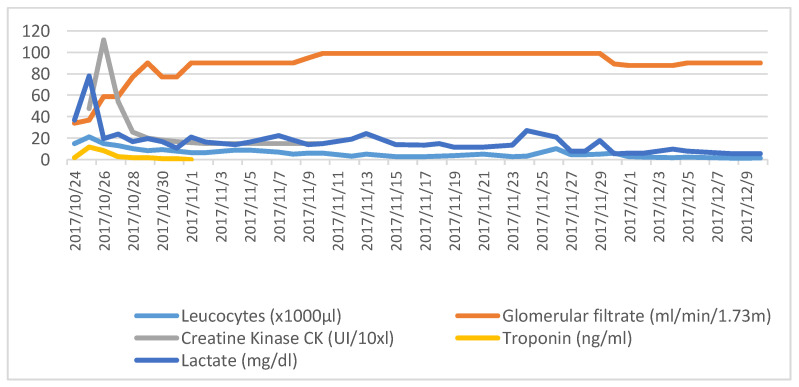
Changes of the main lab parameters solicited during the stay.

The first results obtained from the laboratory were hemoglobin 13.40 g/dL, platelets 567,000 uL, and leucocytes 18,550 uL, of which 94.1% were neutrophils; see also the curves of the measurements in Figure 1.

The parameters of basic coagulation remained stable during her stay. There was a deterioration in the renal function in the beginning, with a minimum glomerular filtrate of 34 mL/min/1.73 m^2^, which subsequently normalized, and with the urea concentration and ionogram (sodium, potassium calcium and magnesium) within a normal range.

Coinciding with the initial respiratory deterioration, the arterial gases obtained had the following characteristics: pH 6.9, pCO 93 mmHg, pO2 100 mmHg, HCO3 20 m Eq/L, base excess −12 m Eq/L, with lactate at 79 mg/dL. These parameters were corrected with invasive mechanical ventilation.

The biochemical analysis underlined a tendency towards hyperglycemia, as well as a high level of transaminases, a peak of troponin (11.74 ng/dL) at 48 h after admission that was related to the spasms, as well as an increase in creatine kinase (CK), which reached 1116 UI/L 72 h after her arrival. She also had toxins in her urine that were positive for cocaine. The PCR and the procalcitonin remained negative in all the tests.

During the admission, other parameters were quantified related to her previous pathology: viral HIV-1 load, not detectable; Hepatitis B Antigen, surface negative; positive Hepatitis B core antibody; Hepatitis B surface antibody quantitative, negative; Hepatitis C IgG antibody, positive; Hepatitis C antibody confirmation, positive; total antibodies HIV, positive. As well as other cell studies: total lymphocytes: 720 uL; lymphoid marker CD3·61.19%, T4 (CD4) 22.77%, T8 (CD8) 35.06%, and CD4/CD8 coefficient 0.65; total CD4 lymphocytes 164/mm^3^; and total CD8 lymphocytes 252/mm^3^.

The comorbidities of the patient, along with the expected complications from a severe illness such as tetanus, led to the sequence of tests performed during the progression, as can be observed in Table 1.

### 2.4. Treatment

The temporal sequence of the drugs administered during the patient’s stay are shown in Table 2.

It should be highlighted that the treatment required a high dose of sedatives and relaxants to control the painful spasms that are typical of the disease. The patient suffered a *Candida tropicalis* colonization that was treated with fluconazole, to which prophylactic co-trimoxazol was added due to a determination of CD4 < 200.

During the admission, an anti-retroviral treatment was administered through a nasogastric probe (Kaletra^®^, Epivir^®^, Intelence^®^, and Fluzcon^®^) and a parenteral nutritional support was provided, which covered the high requirements provoked by the disease. Furthermore, prophylaxis with heparin of low molecular weight and with proton pump inhibitors were administered.

## 3. Discussion

The uniqueness of the case lies on the suite of comorbidities the patient suffered, to which we have to add being an IDU as a defining risk factor. Some time ago, IDUs were identified as a high-risk population who were susceptible to suffering from parenterally acquired diseases (HIV, HCV, HAV, HBV, tetanus, syphilis, and malaria) [5,6,7]. Their susceptibility comes from the nature of the secondary wounds after venipuncture with non-sterilized materials, normally in subcutaneous tissue, which favor the appearance of abscesses and the growth of anaerobic organisms [5]. The IDUs assume a high risk of self-inoculation when they utilize contaminated needles to inject themselves in debilitated tissues [10]. In the United States, during the 2009 to 2017 period, 264 tetanus cases were registered, of which 8% (21 of the cases) involved IDUs [11]. These types of patients tend to have sporadic contact with the health system, so that it is considered that unless they had been vaccinated during childhood, it is highly likely that they are not immunized [12]. Every contact with the health system should be taken advantage of, as any approach to the system could provide an occasion for immunization, which is the most cost-effective manner for reducing the mortality associated with tetanus [13]. In Europe, the tetanus vaccine is recommended for adults starting at the age of 65, but there are 4 countries (Iceland, Ireland, Serbia, and the United Kingdom) that recommend them for adults who belong to risk groups, such as IDUs [14].

For those infected with HIV and HCV, two of the comorbidities present in this case, a permanent state of activation of the immune system has been described, which has been related to the quality of the response to the vaccines. Elevated plasma levels of IL-6, CD14, CD163, and IP10, immediately before vaccination, are inversely related to the immune response developed after the administration of the vaccines against HAV/HBV and the anti-tetanus booster, in patients infected with HCV or HIV [15]. Aside from a lower immunological response, it has been shown that the duration of the seroprotection between boosters is lower in HIV patients [16]. 

For the vaccines currently available for these infectious diseases (HBV, HAV, and tetanus), contradicting reports exist about the immunogenicity of the IDUs. The hepatitis B vaccine, another of the diseases associated with the IDU present in this case, has been available for more than 20 years. However, it has been described that its absorption is very low in IDUs, even lower than 30% [6].

Immunization against tetanus is the only protection against this severe disease. The tetanus vaccine is considered by the WHO as being very safe, also for HIV patients and immuno-depressed individuals [17]. The data from the serological studies show that in order to obtain prolonged protection, at least 5 doses are needed, and this vaccination seems to be less effective in those older than 60 years old due to a lower activity of the cellular immunity mediated by T lymphocytes [6,18,19]. Tetanus antitoxin antibodies titers of at least 0.01–0.2 IU/mL are considered good levels of protection, as determined in serum through a standard ELISA test. However, tetanus cases have been documented in people with antitoxin concentrations higher that these thresholds [5,17]. It has been confirmed that tetanus adopts clinical forms that are more severe in individuals who are not immunized or those with low levels of antibodies when comparing them with individuals with correct immunization, and has also been associated with a higher mortality rate [20].

It is not easy to obtain immunization data of individuals who are in penitentiary centers, and most of the studies are focused on hepatitis B coverage [21]. However, it is known that in the United States, the youth institutionalized in correction centers are sensibly better immunized than the general population [22].

As described, in the present case there were important factors of risk, and the patient had not been correctly immunized either. She was not vaccinated during her admissions to prison or during the monitoring conducted by the Infectious Diseases Unit. This is in agreement with the results from a study conducted in the UK in 2014 with IDUs, in which inadequate levels of antibodies against tetanus were observed in this population, as well as a high portion of cases who had never been vaccinated [7].

The tetanus diagnostic is based on the clinical results. According to the criteria from the WHO, in adults, after an antecedent of a wound, one of the following symptoms should appear for the diagnosis: trismus, risus sardonicus, or painful muscle spasms [20,23]. It is of vital importance to perform an adequate anamnesis with an exhaustive recompilation of the epidemiological antecedents and to know the vaccination state to guide the diagnosis. Complementary tests tend to be conducted to reject other types of neurological and/or otorhinolaryngology pathologies that are part of the differential diagnosis. Given that a diagnostic test for tetanus does not exist, the availability of a fast bedside test (Tetanus Quick Stick, Nephrotek Laboratory, Rungis, France) to clarify the state of immunization of the patient could be of great help [24].

Our patient had the general form of tetanus, which is the most severe and the most frequently found. This form is congruent with the deterioration of the defenses of individuals with HIV, who are at a greater risk of illness, have a greater severity of vaccine-preventable diseases, and higher rates of hospitalization once they become sick.

As for the treatment, 4000 IU of intravenous immunoglobulin were administered, in agreement with traditional recommendations. However, organizations such as the WHO and the CDC argue for the administration of lower doses (500 IU). In the literature reviewed, the most-utilized doses were between 3000 and 6000 IU [5,20,25,26,27,28,29].

Measures were also adopted that involved hemodynamic and especially respiratory support, as well as antibiotic coverage. In relation to this, various studies prioritize the use of metronidazole as opposed to penicillin, given that penicillin has been linked with an increase in the inhibitory effects on neurotransmitters, and therefore with the exacerbation of the disease [7,30]. The use of assisted breathing methods has considerably improved the prognosis of tetanus; nevertheless, the total mortality rate due to tetanus is high even in intensive care units that have a great amount of resources [31,32]. A higher mortality rate has been described for patients with general tetanus, high fever, and tachycardia and a period of incubation of less than 7 days [33]. The figures oscillate between 10 and 70%, depending on the treatment, age, and the prior state of health [2]. 

The rest of the treatments administered for the control of symptoms were congruent with what had been already published, and the use of the antibiotics was well-suited for the complications and the antibiograms obtained.

The general form of tetanus is associated with long hospital stays with the need for ICU care during most of the stay. The stay in the ICU in the present case described lasted 47 days. Nicolai et al. in 2015 referred to stays lasting 32 days in critical care units for patients who survived [18]. On the contrary, there are studies with shorter stays, such as in a Turkish series of 43 cases published in 2003, which described an average stay of 14 days [31].

## 4. Conclusions

Tetanus is a severe disease that is preventable with safe vaccines. There are more vulnerable collectives who are not immunized or who have immunization insufficiency, for whom any contact with the health system should be taken as an opportunity to improve the vaccination status; the disease also entails a greater morbi-mortality for them.

The clinical suspicion when dealing with symptoms compatible with tetanus is fundamental and should be supported with the knowledge of the previous vaccination state, the patient’s personal antecedents, and if possible, the bedside determination of the immunization state through the use of a fast test.

The diagnosis of tetanus, although infrequent in the developed world, should not be underestimated or forgotten. The notoriety of the present case comes from the set of risk factors and the comorbidities of the patient—these being an IDU having both HIV and HCV, the opportunities missed for vaccination after passing through different institutions, and, especially, that she survived tetanus.

## Figures and Tables

**Table 1:** Complementary tests performed during the hospital stay.

Date	Test	Result
24/10/17	CT scan, cervical and cranial	Without significant findings
	2nd CT scan cranial (due to spasms-convulsions)	Without significant findings
	Potable chest radiography	Bibasal infiltrates
	Spinal tap. CSF	Glucose 117; Total Proteins 41.9; VDRL Negative.
	CSF culture	Herpes I (PCR) Negative, Herpes II (PCR) Negative, *Cryptococcus neoformans* (India ink) negative.
	ECG	No acute re-polarization alterations
25/10/17	ECG	No acute re-polarization alterations
	EMG	Lack of activity, probable relation with muscle relaxant
30/10/17	1st Bronchoscopy	Tracheostomy Aspiration of purulent secretions
02/11/17	Pulmonary ECO	Pulmonary condensation, right
07/11/17	Echocardiogram	LVEF normal (75–80%); no alterations
08/11/17	CT scan chest and abdomen-pelvic with contrast (due to fever peak)	Complete atelectasis of the RLL. Blockage of the main right bronchus and the RLL suggestive of pneumonia/atelectasis due to aspiration. Opacity in RUL and LLL due to bronchopneumonia.
	2nd Bronchoscopy	Aspiration of purulent secretions.
23/11/17	EEG	No pathological findings
30/11/17	EMG	Sensory-motor polyneuropathy, mixed, with distal and symmetric distribution, and severe degree, with presence of active denervation signs of the lesion. Spasms are not evidenced.
5/11/17	Blood cultures Catheter culture Bronchial aspirate culture	*Candida tropicalis* *Klebsiella pneumoniae* producer of ESBLs

CT scan: computerized tomography; CSF: cerebrospinal fluid; VDRL: Venereal Disease Research Laboratory for syphilis; ECG: electrocardiogram; EMG: electromyogram; ECO: echography; LVEF: left ventricle ejection fraction; RLL: right lower lobule; LLL: left lower lobule; RUL: right upper lobule; EEG: electroencephalogram; ESBLs: extended spectrum β-lactamases.

**Table 2:** Treatments administered during the hospital stay.

Treatment	Date (Year 2017)																							
	Oct 25	Oct 27	Oct 29	Nov 1	Nov 2	Nov 4	Nov 6	Nov 8	Nov 10	Nov 12	Nov 14	Nov 16	Nov 18	Nov 20	Nov 22	Nov 24	Nov 26	Nov 28	Nov 30	Dec 2	Dec 4	Dec 6	Dec 10	Dec 12
^a^ Midazolam 250 mg/250 mL G5% (mL/h)	20 *	20 *	20 *	15	15	15	15	15	0 *	*	20 *	20	*	*	20	*								
^a^ Propofol 2% (mL/h)	10 *	10 *	10	10 *	10	10	10	10	*	*	10 *	10	10	*										
^a^ Magnesium sulfate 15% 10 mL (3 amp/100 mL SF) (mL/h)	4	4	4	4	4	*	4 *	4	4	4	4	4	4	4	4	4	4	4	4	4	4	4	0	0
^a^ Cisatracurium (50 mg/100 mL SF) (mL/h)	8 *	8 *	8	4 *	4 *	4 *	4	4 *	8	8	8	6	4	4	4									
^a^ Morphine 1% (60 mg/100 mL SSF 0.9%) (mL/h)	4	4	4	4	4	4	4	4	4	*	4	4	*	*	*	4								
^a^ Noradrenaline (10 mg/100 mL SF) (mL/h)	8	8	6	8	4	4	4	6	5	4	4	2	2	3	2	2								
^a^ Labetalol (100 mg/20 mL) (mL/h)															15	15								
^a^ Baclofen 23 mg/day																								
^b^ Piperacillin-Tazobactam 4 g/6 h																								
^b^ Levofloxacin 500 mg/12 h																								
^b^ Metronidazole 500 mg/8 h																								
^b^ Linezolid 600 mg/12 h																								
^b^ Trimetoprim/sulfamehtoxazole 800/160 mg/24 h																								
^b^ Meropenem 1 g/8 h																								
^c^ Boostrix^®^ pre-loaded syringe																								
^c^ TIG 4000 UI																								
^d^ Temperature (°C)	38	37	38	36	38	37	38.5	36	36.5	36.4	36.7	36.7	36.5	36.4	36.7	37	38	39	38.5	38	37	36.5	36.6	36.5

^a^ Symptom treatment against tetanus; ^b^ antibiotics; ^c^ vaccine (Td) and tetanus immunoglobulin (TIG). ^d^ Temperature progression. In grey: days of intervention. * Extra doses of treatment; The background highlights which day the treatment is necessary.
